# Performance and mechanism of waste engine oil in the regeneration of aged asphalt from different perspectives

**DOI:** 10.1371/journal.pone.0329203

**Published:** 2025-07-24

**Authors:** Yi Wang, Jin Wang, Wei Cui, Hongwei Wang, Fan Zhang, Fayong Yang

**Affiliations:** 1 College of Technology and Engineering, Xi’an Kedagaoxin University, Xi’an, China; 2 Xi’an Highway Research Institute Co., Ltd., Xi’an, China; Shandong University of Technology, CHINA

## Abstract

To expand the utilization pathways of waste engine oil (WEO) and address the issues arising from road aging, this study investigates the regeneration effect of WEO as a rejuvenator for aged asphalt. The performance degradation of asphalt under different aging durations was analyzed, and a predictive model correlating aging time with performance decline was developed. Then, the influence of different dosages of WEO on the regeneration of aged asphalt was examined, and the regenerative mechanism in aged asphalt was explored through microscopic analysis. Furthermore, the molecular simulation was employed to elucidate the molecular-level interactions between the rejuvenator and aged asphalt. The results showed that as aging time increases, the rotational viscosity at 135°C increases with aging time, rising by 8.9% to 40.9%, indicating the changes in the fitting equations of various predictive models. Also, it was found that with an optimal dosage of 2%, the rejuvenator can significantly restore the basic performances of aged asphalt. Micromorphological analysis revealed that the surface of aged asphalt exhibited an uneven and groove-like structure, while the surface of recycled asphalt became shallower and more uniform. Four-component analysis showed that the proportion of light components in aged asphalt decreased, while the proportion of gel-like components increased. The addition of the rejuvenator effectively replenished the light oil components lost during aging. Additionally, there was a significant reduction of carbonyl and sulfoxide groups in aged asphalt. Molecular simulations revealed that the diffusion ability of light components in aged asphalt was weakened, while the Ar and S diffusion coefficients increased by 861.4% and 108%, respectively, indicating that the diffusion ability of these components was notably improved. Consequentially, the application of WEO as a rejuvenator in asphalt modification offers significant economic and environmental benefits.

## 1. Introduction

With the continuous advancement of strategies to build robust transportation infrastructure, China’s road network has been substantially expanded, with the total length of maintained roads exceeding 6 million kilometers and still growing [1]. Despite these developments, asphalt pavements inevitably suffer from prolonged exposure to traffic loads and environmental factors, which accelerate asphalt aging and induce pavement distress. Annually, more than 100 million tons of waste asphalt mixtures are generated during road maintenance [2], and improper disposal of these materials results in considerable resource wastage and environmental concerns. Consequently, the effective management and recycling of waste asphalt mixtures have become critical challenges for road maintenance and sustainability efforts. The primary cause of asphalt aging is the oxidation and volatilization of light components, leading to a significant increase in viscosity and brittleness of aged asphalt compared to fresh asphalt, with viscosity approximately doubling post-aging [3].

Conventionally, rejuvenators formulated from petroleum-derived aromatic oils, alkanes, and light oils are applied to restore the properties of aged asphalt by replenishing lost components. While these rejuvenators can improve pavement performance, their production and application processes contribute substantially to carbon emissions [4]. Moreover, these petroleum-based rejuvenators tend to degrade under high temperatures, limiting their long-term effectiveness to the early stages of asphalt aging. More importantly, reliance on non-renewable fossil fuels contradicts the growing global emphasis on sustainable development and carbon neutrality. In response to evolving environmental policies and the “dual carbon” (carbon peaking and carbon neutrality) goals, traditional petroleum-based rejuvenators are progressively being phased out [5–7].

In parallel, the rapid growth of automobile ownership has led to an increase in used motor oil generation during vehicle maintenance, estimated at approximately 1.58 × 10⁹ liters annually in China alone [8]. However, the recycling rate remains low, with only about 20% of used engine oil being properly reclaimed [9–11]. Improper disposal of such a vast quantity of waste oil poses severe environmental threats, including soil and water pollution [12]. Therefore, the development of effective strategies for the environmentally sound disposal and resource recovery of used engine oil has become a vital research focus.

Recent studies have highlighted the potential of hydrocarbon-rich waste oils in rejuvenating aged asphalt [13–15]. Waste-derived oils—including waste engine oil (WEO), waste cooking oil (WCO), and their residues—have been evaluated for their efficacy as rejuvenators. For example, Zhang et al. [16] demonstrated that bio-oil extracted from waste wood oil, at an optimal dosage of 15%, significantly enhanced the rutting and fatigue resistance of aged asphalt by over 150%. Abdullah et al. [17] reported that WEO and WCO can restore asphalt performance at optimal dosages of 7% and 13%, respectively. Muhammad et al. [18] confirmed that WEO improves fatigue resistance and thermal cracking performance, although excessive addition may reduce rutting resistance. Other studies, including those by Sun et al. [19], Wang et al. [20], Peng et al. [21], and Zhang et al. [22], further substantiate the effectiveness of various bio-oils in restoring aged asphalt properties, with performance depending on oil type and dosage.

Additionally, Sun et al. [23] prepared a waste cooking oil-based rejuvenator that restored aged asphalt properties to near-original levels at 5% dosage, supported by both macro- and micro-scale analyses. Liu et al. [24] noted that adding WEO alongside modifiers like SBS and EVA improves storage stability but cautioned about its adverse effects on high-temperature performance when overdosed. Yan et al. [25] demonstrated that vegetable oils such as tung oil and WCO enhance the overall road performance of recycled asphalt mixtures. Conversely, Magdy et al. [26] found that WEO alone can disrupt asphalt’s molecular structure and mechanical balance, whereas combining WEO with rubber powder yields superior performance.

In summary, despite advances in bio-oil-based rejuvenators, the comprehensive understanding of WEO’s regeneration performance and mechanisms remains limited. This study aims to systematically investigate the effects of WEO on the physical and chemical properties of aged asphalt and elucidate its regeneration mechanisms. By doing so, it seeks to provide scientific guidance for the sustainable utilization of waste oils in asphalt rejuvenation, advancing resource recycling and supporting environmental protection and carbon neutrality goals in the transportation sector.

## 2. Materials and methods

### 2.1. Materials

#### 2.1.1. Asphalt.

In this study, Karamay 70# matrix asphalt (MA) was selected. The performance tests were conducted according to the “Standard Test Methods of Bitumen and Bituminous Mixtures for Highway Engineering” (JTG E20-2011) [27]. The measured results are presented in Table 1.

**Table 1:** Performance index of 70# MA.

Items	Units	Measured value	Technical Specification	Test method [27]
Penetration (25°C, 100g, 5s)	0.1mm	68.1	60 ~ 80	T 0604–2011
Ductility (10°C)	cm	50.8	≥25	T 0605–2011
Softening point	°C	81	46.0	T 0606–2011
Viscosity (135°C)	Pa·s	0.773	–	T 0625–2011
RTFOT (165°C, 5h)				
Mass Change	%	0.41	±0.8	T 0609–2011
Residual penetration ratio	%	69.3	≥61.0	
Residual ductility (15°C)	–	11.3	≥15.0	

#### 2.1.2. WEO.

WEO was sourced from a 4S store in Xi’an. Due to the presence of metal debris in the recycled oil, it was necessary to decontaminate the WEO before use [28]. The main components of WEO are shown in Table 2

**Table 2:** Composition of WEO Components.

Type	Main components	Additives	Impurity
WEO	Base oil	Anti-aging agent, dispersant, emulsifier, etc.	Metal shavings, dust, moisture, etc.

**Fig 1:**
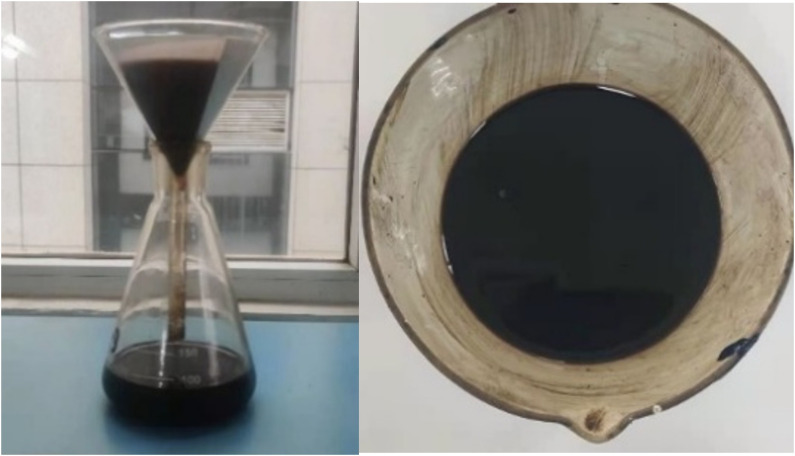
WEO filtration device.

Due to the complex composition of the recycled WEO, the WEO is first filtered to remove impurities and metal particles. The filtration equipment employed for this process as shown in Fig 1. WEO is filtered once in the filtering device for about 10 minutes to obtain a relatively clean WEO.

### 2.2. Methods

**Fig 2:**
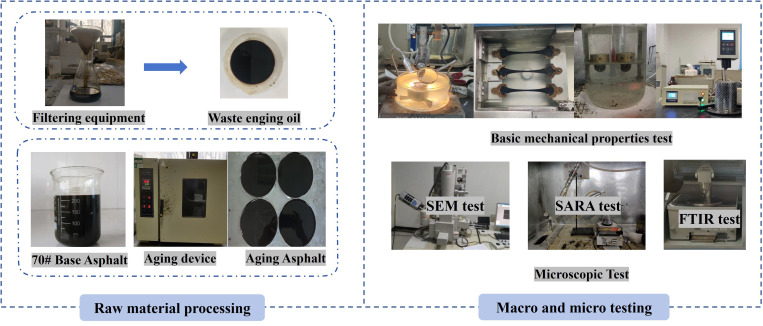
Experimental process.

In this study, both macro (penetration, softening point, ductility, and viscosity) and micro performance tests were conducted following the Chinese National Standards “Standard Test Methods of Bitumen and Bituminous Mixtures for Highway Engineering” (JTG E20-2011). The flowchart outlining the research process is shown in Fig 2.

(1) Basic performance test

Basic performance tests (penetration, softening point, ductility, and viscosity) were performed following the requirements of protocols T 0604–2011, T 0605–2011, and T 0606–2011. In this case, the penetration test is conducted at a temperature of 25°C, the ductility test at 10°C, and the viscosity test at 135°C.

(2) Thin Film Oven Test (TFOT)

To assess the effectiveness of WEO in restoring asphalt performance, different asphalts were subjected to testing and analysis through a TFOT. The aging test was conducted following the T 0609–2011 protocol in JTG E20-2011, with an aging time of 5 hours at a temperature of 163°C.

(3) SARA test

The performance of asphalt is significantly impacted by the amount of the four fractions (S(saturate), A(aromatic), R(resin), A(asphaltene), SARA) of asphalt. The SARA test was carried out according to the protocol T 0618–1993.

(4) Scanning electron microscope (SEM) test

To analyze the reasons for the changes in asphalt performance after the addition of WEO, SEM observations were conducted on different asphalts. The scanning electron microscope instrument used for the test was the S-4800 model manufactured by JEOL, Japan, as depicted in Fig 1. Before observing the asphalt samples, a gold spray treatment was applied, and the magnifications were 200 × , 1000 × , and 1500 × .

(5) Fourier transform infrared spectrum test

Changes in functional groups are key parameters for analyzing material modification and performance enhancement. In this study, a Varian 600-IR series Fourier Transform Infrared Spectrometer (FTIR) was used to test the spectral range of 4000–500 cm ⁻ ¹, as shown in Fig 2.

## 3. Results and discussion

### 3.1. Effect of aging time on asphalt properties

**Fig 3:**
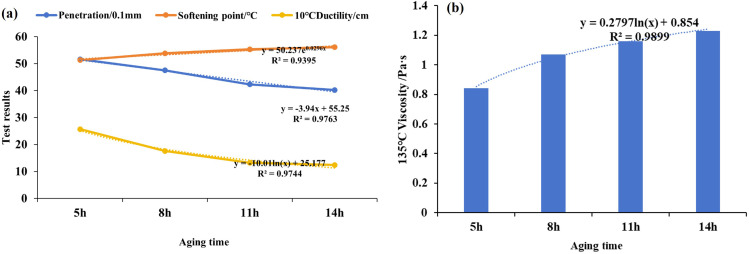
Basic properties of asphalt at different aging times. (A) Changes in the three main indicators; (b) Viscosity change).

To further analyze the effect of aging time on the regeneration of asphalt using WEO, the basic performance of matrix asphalt at different aging times was examined according to the Chinese national standard “Standard Test Methods of Bitumen and Bituminous Mixtures for Highway Engineering”(JTG E20-2011). The test results are presented in Table 3 and Fig 3.

**Table 3:** Results of matrix asphalt performance tests under different aging times.

Aging time	Penetration/0.1 mm	Softening point/°C	Ductility/cm	135°C Viscosity/Pa·s
5h	51.6	51.3	25.7	0.842
8h	47.5	53.8	17.6	1.072
11h	42.3	55.3	13.2	1.161
14h	40.2	56.1	12.4	1.230

As shown in Fig 3(a), compared to the original matrix asphalt, the degree of penetration decreases with increasing aging time, exhibiting a reduction of 24.2% to 40.1%. This represents the hardening of the asphalt and a reduction in temperature sensitivity. Additionally, with increasing aging time, the softening point of the asphalt shows a consistent upward trend, increasing by 0.98% to 10.4%, indicating improved high-temperature performance. Conversely, the ductility of the asphalt decreases significantly, with a reduction of 68.3% to 84.7%, suggesting a greater loss of low-temperature performance due to heat aging. As observed in Fig 3(b), the rotational viscosity at 135°C increases with aging time, rising by 8.9% to 40.9% compared to the original matrix asphalt, indicating that the asphalt becomes more viscous as aging progresses.

Various regression models—linear, exponential, logarithmic, and polynomial—were applied to explore the relationship between aging time and asphalt performance. The results, summarized in Table 4, indicate that the optimal fitting method differs across performance indicators. Specifically, the penetration index is best described by a linear model, whereas ductility and viscosity at 135 °C are more accurately represented by logarithmic models. In contrast, the softening point is most suitably fitted using an exponential model. The optimal fitting equations, along with their corresponding Pearson correlation coefficients, are presented in Table 4. These findings confirm that all selected models achieve high predictive accuracy, with correlation values exceeding 90%, suggesting that the aging performance of asphalt can be reliably predicted using these regression approaches.

**Table 4:** Prediction formulas for fitting different indicators.

Performance indicators	Formula	R^2^
Penetration	y=−3.94x+55.25	0.9763
Softening point	$y=50.237e^{0.0296x}$	0.9395
Ductility	y=−10.01lnx+25.177	0.9744
Viscosity	y=0.2797lnx+0.854	0.9899

### 3.2. Effect of WEO blending on the basic properties of aged asphalt

**Fig 4:**
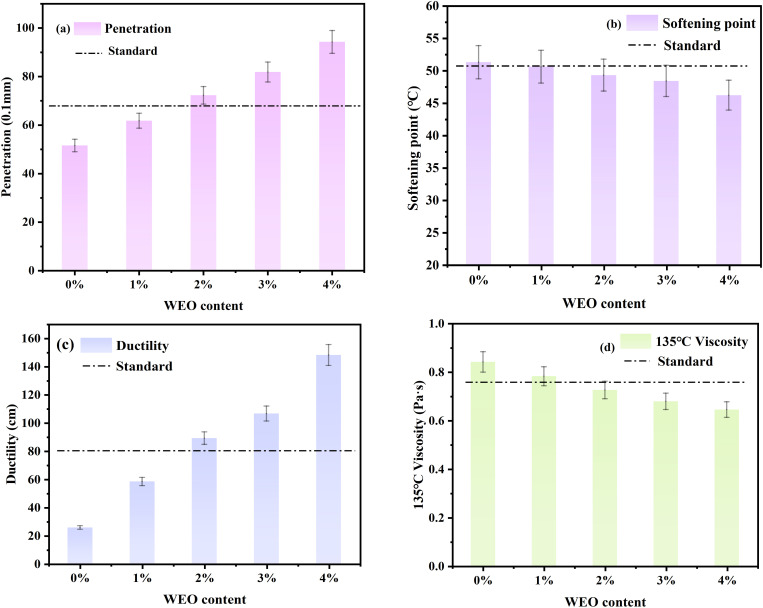
Basic performance results of aged asphalt regenerated from WEO. (A) Penetration; (B) Softening point; (C) Ductility; (D) 135°C Viscosity.

The regeneration of aging asphalt after 5 hours heating was conducted using WEO as the regenerator, with dosages of 1%, 2%, 3%, and 4%. The test results are presented in Table 5 and Fig 4.

**Table 5:** Test results of basic performance of aged asphalt regenerated from WEO.

Asphalt type	Penetration/0.1 mm	Softening point/°C	Ductility/cm	135°C Viscosity/Pa·s
70#	68.1	50.8	81	0.773
TFOT (Aged asphalt)	51.6	51.3	25.7	0.842
1%WEO+Aged asphalt	61.8	50.6	58.4	0.783
2%WEO+Aged asphalt	72.3	49.3	89.2	0.726
3%WEO+Aged asphalt	81.9	48.4	106.7	0.679
4%WEO+Aged asphalt	94.3	46.2	148.3	0.645

As shown in Table 5 and Fig 4(a), WEO demonstrates a clear ability to restore the penetration index of aged asphalt. With increasing WEO content, the penetration index improves by 19.8% to 82.8% compared to that of the aged asphalt, indicating that WEO effectively rebalances the material’s hardness and softness. A dosage between 1% and 3% is sufficient to restore the penetration index to a level comparable to that of the original matrix asphalt.

Table 5 and Fig 4(b) also indicate that WEO facilitates the recovery of the softening point. However, the variation in this index remains relatively modest. As the WEO dosage increases, the softening point of aged asphalt exhibits a slight downward trend, decreasing by 1.4% to 9.9%. Compared to its effect on the penetration index, WEO’s influence on the softening point is less pronounced. Nevertheless, a dosage between 0% and 2% is adequate to restore the softening point to that of the matrix asphalt.

As illustrated in Table 5 and Fig 4(c), the addition of WEO markedly enhances the ductility of aged asphalt, with improvements ranging from 127.2% to 477.0%. This substantial increase highlights WEO’s effectiveness in improving the low-temperature performance of aged asphalt. Specifically, a WEO content of 1% to 3% can fully restore ductility to the level of the matrix asphalt.

Furthermore, Table 5 and Fig 4(d) show that the rotational viscosity of aged asphalt at 135 °C decreases with increasing WEO content. Although the effect of WEO on viscosity is less significant than its impact on penetration or ductility, it still results in a reduction of 7.0% to 23.4%. Within a dosage range of 0% to 2%, the viscosity of the rejuvenated asphalt can be effectively restored to its original level, thereby improving its workability during mixing and application.

In summary, the results indicate that WEO is effective in regenerating aged asphalt, with varying degrees of influence on different performance indicators. The most significantly affected properties are penetration and ductility, followed by rotational viscosity at 135°C, while the softening point exhibits the least change. Considering the overall impact on these indicators, the optimal WEO dosage as a rejuvenating agent is determined to be 2%.

### 3.3. Mechanism analysis of aging asphalt regenerated from WEO

#### 3.3.1 Asphalt surface morphology analysis.

**Fig 5:**
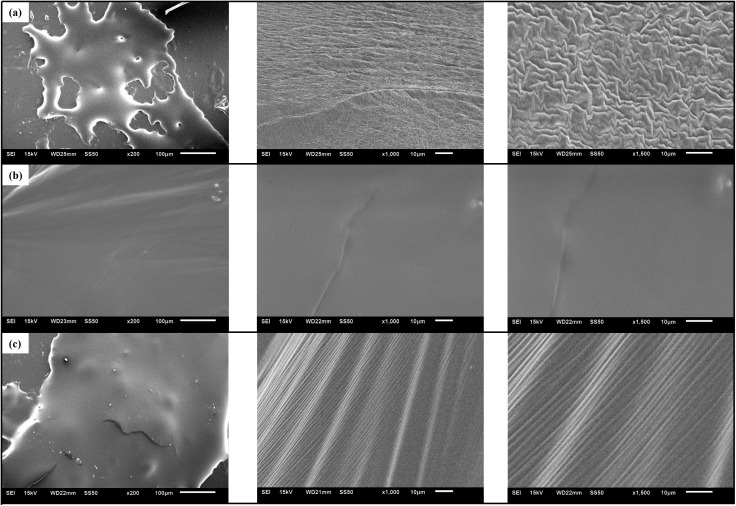
Microscopic morphology of different asphalts (200 × , 1000 × , and 1500×). (A) Matrix asphalt; (B) Aged asphalt; (C) Recycled asphalt (2).

SEM tests were performed on the original matrix asphalt, aged asphalt, and recycled asphalt (containing 2% WEO) at magnifications of 200 × , 1000 × , and 1500 × , respectively. The objective was to analyze surface morphology and establish its relationship with asphalt performance. The SEM test results are presented in Fig 5.

As illustrated in Fig 5, the surface of the unaged matrix asphalt exhibits a relatively smooth morphology at low magnification, which becomes increasingly uniform at higher resolutions. Upon aging, solid precipitates emerge on the surface, likely attributable to the aggregation or increased visibility of asphaltenes. At higher magnifications, continuous and irregular grooves are observed, presumably caused by the volatilization of light fractions, thereby accentuating the presence of asphaltenic structures. Following the incorporation of WEO, the pronounced surface protrusions characteristic of aged asphalt are substantially reduced, resulting in a more homogeneous and saturated surface texture. Although residual grooves remain visible under high magnification, their significantly reduced depth compared to the aged specimen indicates an improvement in surface integrity and morphological stability.

In summary, the microstructural characteristics of asphalt surfaces differ markedly across aging and rejuvenation states. The original asphalt surface is smooth and uniform, with high-magnification images resembling a mirror-like texture. In contrast, aged asphalt exhibits surface roughness and irregular grooves, which are likely linked to its deterioration in macroscopic properties. The recycled asphalt surface, while still containing some grooves, presents a much more uniform and compact morphology.

This enhanced microstructure likely contributes to the recovery of macroscopic performance indicators such as penetration, ductility, and viscosity. Therefore, the improved surface continuity observed at the microscopic level offers compelling evidence of the correlation between morphological refinement and performance restoration in rejuvenated asphalt systems.

#### 3.3.2. SARA test analysis.

**Fig 6:**
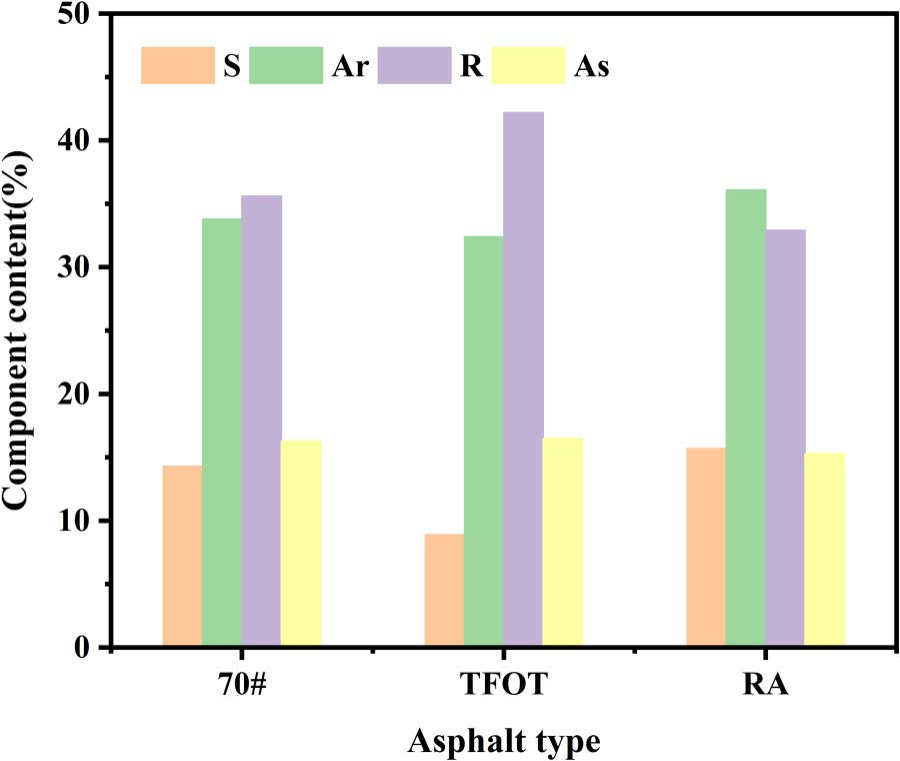
SARA test results for different types of asphalt.

To analyze the aging mechanism of asphalt and the regeneration mechanism of WEO in rejuvenating aged asphalt, a SARA (S(saturate), A(aromatic), R(resin), A(asphaltene), SARA) test was conducted on matrix asphalt, aged asphalt, and WEO-regenerated aged asphalt, focusing on component migration. The test results are presented in Table 6 and Fig 6.

**Table 6:** SARA test results of different asphalt.

Type of asphalt	S (%)	Ar (%)	R (%)	As (%)
70#	14.3	33.8	35.6	16.3
TFOT	8.9	30.4	42.2	18.5
RA	15.7	36.1	32.9	15.3

As shown in Table 6 and Fig 6, after aging, the proportion of saturated and aromatic in the asphalt decreases significantly, while the proportion of resin and asphaltenes increases. Compared to the matrix asphalt, the saturated and aromatic components decreased by 37.8% and 10.1%, respectively, while the proportion of resin and asphaltenes increased by 18.5% and 13.5%, respectively. After the addition of WEO, there is a significant recovery in the content of the saturated and aromatic, while the content of resin and asphaltenes decreases. This suggests that the addition of WEO helps restore the light components that were lost during aging, which are crucial for the fluidity of the asphalt. The saturated and aromatic, being primarily hydrocarbons, play a key role in determining the mobility of the asphalt. The component order in terms of content distribution is as follows: RA > 70# > TFOT, which aligns with the results obtained for the 135°C viscosity test (RA > 70# > TFOT).

#### 3.3.3. Infrared spectroscopy test analysis.

**Fig 7:**
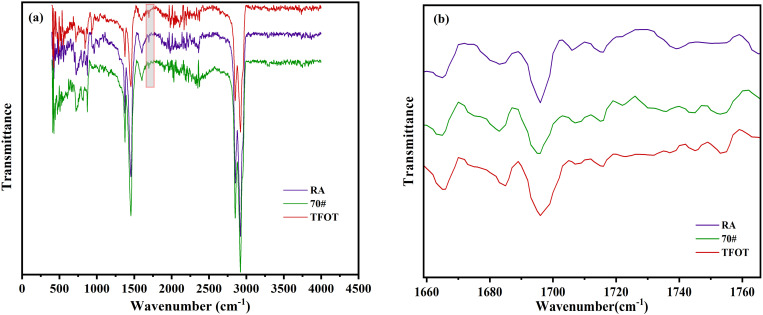
FTIR test results of asphalt. **(A) 400 cm**^**-1**^ ~ 4000 cm^**-1**^; (B) 1600 cm^**-1**^ ~ 1800 cm^**-1**^.

To further investigate the regeneration mechanism of WEO on aged asphalt, FTIR tests were conducted on the original matrix asphalt, aged asphalt, and recycled asphalt. By analyzing the changes in functional groups, the study aims to accurately assess the regeneration effect of WEO on aged asphalt. The test results are presented in Fig 7.

As observed in Fig 7, the absorption peaks of the different asphalt samples appear at approximately the same wavenumbers, with no significant new peaks emerging. This indicates that both the aging and regeneration processes are primarily physical in nature, rather than involving major chemical structural transformations. However, variations in the intensity and area of specific absorption peaks suggest differences in the concentration of functional groups, which in turn influence the macroscopic properties of the asphalt. Notable absorption peaks are located at 1250 cm ⁻ ¹, 1450 cm ⁻ ¹, 2850 cm ⁻ ¹, and 2920 cm ⁻ ¹, corresponding respectively to aromatic C–H in-plane bending (1250 cm ⁻ ¹), C–H deformation vibrations (1450 cm ⁻ ¹), and symmetric and asymmetric stretching vibrations of methyl groups in aliphatic C–H bonds (2850 cm ⁻ ¹ and 2920 cm ⁻ ¹). After aging, the intensities of these peaks decrease, indicating a reduction in the hydrocarbon content of the asphalt, primarily due to the volatilization of light components. The introduction of WEO helps to restore the intensity of these peaks, suggesting that it effectively replenishes the light fractions lost during aging.

From the SARA analysis, asphalt is known to be composed mainly of asphaltenes, resins, aromatics, and saturates, with its flow characteristics and mobility largely determined by the proportion of light components, which are predominantly hydrocarbons. As shown in Fig 7(b), an additional peak at 1695 cm ⁻ ¹ becomes more pronounced, especially in aged asphalt. This peak corresponds to carbonyl (C = O) stretching vibrations, reflecting an increase in oxygen-containing polar functional groups formed during oxidative aging. This observation is consistent with previous research findings.

The decrease in aliphatic hydrocarbon peaks and the emergence of the carbonyl peak directly reflect the molecular-level changes that compromise asphalt flexibility and workability. The partial recovery of these peaks following WEO addition supports the restoration of ductility and penetration values, thereby establishing a direct micro–macro correlation between chemical structure recovery and macroscopic performance improvement.

### 3.4. Molecular dynamics simulation analysis

**Fig 8:**
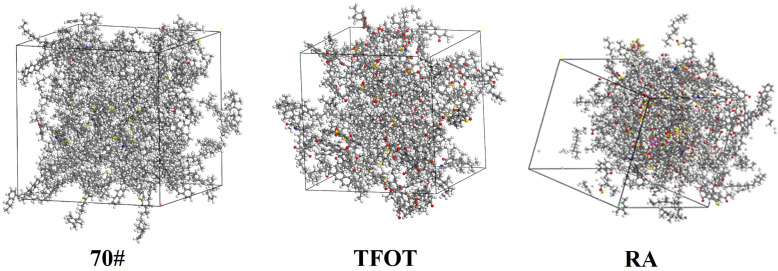
Different asphalt modeling.

In this study, molecular dynamics (MD) simulations of asphalt were performed using Materials Studio 2019. The molecular model was constructed based on the results of a four-component experimental scheme. To ensure structural stability, the model underwent an annealing process prior to simulation. The COMPASS force field was employed to represent the molecular interactions within the asphalt system, and the simulations were conducted under the NVT ensemble. The forcite analysis module was utilized to carry out the dynamic analysis. The diffusion coefficient was selected as a key indicator to evaluate the rejuvenation potential of waste engine oil (WEO) on aged asphalt. The interaction mechanism between WEO and aged asphalt was explored from a molecular-level perspective, providing theoretical insights into the rejuvenation effect. Extensive studies have identified four primary components in asphalt, and numerous representative models have been developed for each of these components. Typically, 12 molecules are utilized for constructing asphalt molecular models [29], and the resulting asphalt model is depicted in Fig 8.

**Fig 9:**
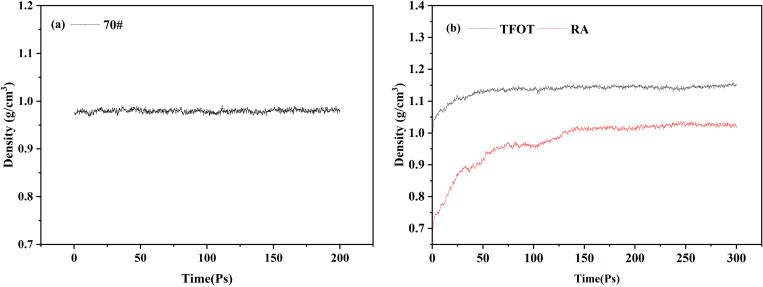
Simulation of asphalt density. (A) 70#; (b) Asphalt before and after recycling.

To ensure the accuracy and stability of the model, the density simulation results are employed to validate the specific test outcomes, as illustrated in Fig 9 and clearly shown in Fig 9. As observed, the density of each asphalt sample stabilizes after approximately 150 ps, indicating that the model has reached equilibrium. Analysis of the 70#, TFOT, and RA simulations reveals stable densities of 0.97 g/cm^3^, 1.14 g/cm^3^, and 1.01 g/cm^3^, respectively. These values closely align with the actual densities of asphalt, suggesting that the model accurately reflects the asphalt aging and regeneration processes [30].

**Fig 10:**
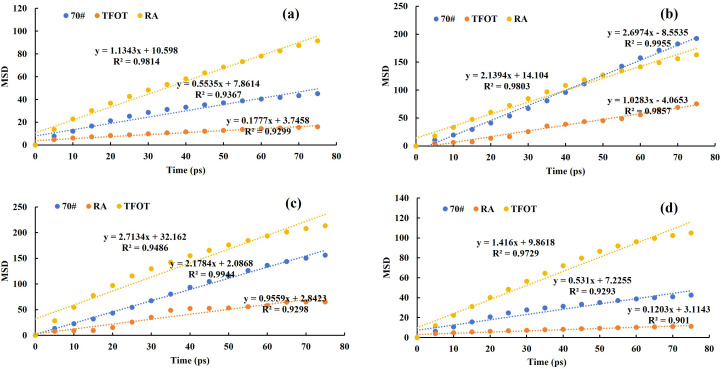
MSD simulation results in asphalt fractions. (A) Ar; (B) S; (C) As; (D) R.


The mean square displacement (MSD) represents the average of the squared molecular displacements within the system at any given time 𝑡, with its calculation method outlined in Equation (1). The diffusion coefficient is numerically approximated as 1/6 times the slope of the MSD-time relationship curve. A larger diffusion coefficient indicates a stronger molecular diffusion ability, as described in Equation (2) [31]. Molecular dynamics simulations of the aforementioned asphalt model were conducted using the mean square displacement method, with the analysis time set to 80 ps. The MSD fitting results, presented in Fig 10, demonstrate that the Pearson correlation coefficients for the MSD of each component in the different asphalt samples exceed 90%, indicating a high degree of fitting accuracy.

$$MSD(t)=\frac{1}{N}\sum_{i=1}^{N}{\left[r_{i}(t)-r_{i}(0)\right]}^{2}$$ (1)

$$D=\frac{1}{6}\lim\frac{d}{dt}\sum_{i=1}^{N}{\left[r_{i}(t)-r_{i}(0)\right]}^{2}=\frac{A}{6}$$ (2)

**Fig 11:**
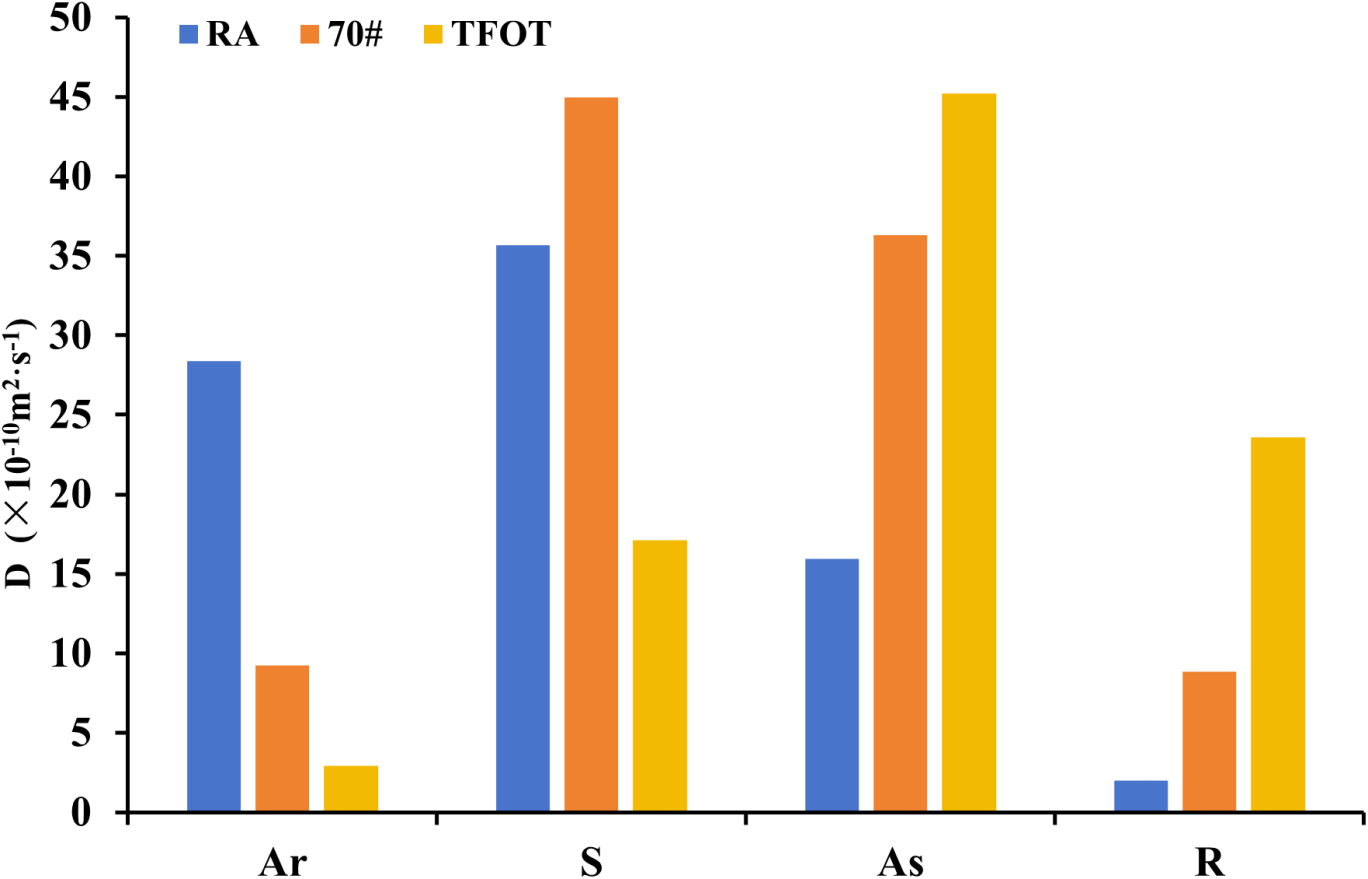
Diffusion coefficients of asphalt components.

Using Equation (2), the diffusion coefficients of each component for matrix asphalt, aged asphalt, and recycled asphalt were calculated. The results are presented in Fig 11. From the test results, it is evident that the diffusion coefficients for 70# matrix asphalt, specifically Ar and S, were 9.23 × 10 ⁻ ¹⁰ m^2^/s and 44.95 × 10 ⁻ ¹⁰ m^2^/s, respectively. After aging, the diffusion ability of Ar and S decreased to 2.95 × 10 ⁻ ¹⁰ m^2^/s and 17.14 × 10 ⁻ ¹⁰ m^2^/s, indicating a reduced activity of the lighter components in the asphalt, which aligns with the macroscopic viscosity test observations. Additionally, the reduction of the lighter components in the four component is consistent with the simulation results. When comparing the diffusion coefficients of aged asphalt with those of asphalt rejuvenated using WEO, the Ar and S diffusion coefficients increased by 861.4% and 108%, respectively. This suggests that WEO can effectively restore the performance of aged asphalt by enhancing the mobility of its lighter components. The simulation results thus confirm the feasibility of using WEO as a rejuvenating agent for aged asphalt.

### 3.5. Analysis of economic and environmental benefits of asphalt regeneration from WEO

Due to the regulatory control over WEO recycling by relevant state departments, specialized institutions are required for its processing. The price of WEO varies depending on the type, with darker oils generally priced lower (approximately 3–4 yuan per pound) and lighter oils priced higher (around 4–5 yuan per pound). On average, the cost of WEO is between 1,800–2,300 yuan per ton. For this study, we assume a price of 2,100 yuan per ton of WEO. Based on trial calculations, one ton of WEO can regenerate approximately 33.3 tons of 70# asphalt. The unit price of AH-70 asphalt is 2,300 yuan per ton. Therefore, the cost of 2,100 yuan per ton of WEO can generate a revenue of approximately 76,600 yuan. After excluding the labor and energy costs associated with the regeneration process, the net profit is estimated to be around 65,000 yuan. This demonstrates that the use of WEO as a rejuvenating agent offers significant economic benefits.

Traditional methods of WEO disposal include regeneration, chemical purification, and use as fuel. While these methods maximize the utility of WEO, they often involve complex processes or present significant environmental challenges. For example, the regeneration process typically requires multiple stages, such as distillation, fractionation, filtration, and refining, often followed by the addition of other components. Each of these stages demands considerable energy input, leading to higher carbon emissions throughout the entire lifecycle. The preparation stage, in particular, involves multiple processes that contribute to both energy consumption and environmental pollution. Furthermore, the complexity of these processes can affect the economic feasibility of regeneration. In contrast, using WEO as a rejuvenating agent for waste asphalt aligns with the principles of waste-to-resource, in line with China’s current environmental policies. This practice not only supports sustainable development but also contributes to the achievement of the country’s dual-carbon goals in the engineering industry.

## 4. Conclusions

This study investigated the use of WEO as a rejuvenator for aged asphalt, analyzing the effect of aging time on asphalt performance, conducting a systematic evaluation of the basic performance of WEO-regenerated asphalt, and exploring the regeneration mechanism of WEO. Finally, the economic and environmental benefits were assessed. The main findings are as follows:

(1) Aging significantly poses a negative effect on the performance of asphalt, leading to hardening and thickening. A comparison of different fitting models indicates substantial differences in the way various performance indicators change over time.
(2) WEO is an effective regenerator for aging asphalt. It restores various performance indicators to varying degrees, with the most pronounced effects observed on penetration and ductility, followed by 135°C viscosity and softening point. The optimal dosage of WEO for regeneration is 2%.
(3) The surface morphology of matrix asphalt is smooth, while aged asphalt displays uneven grooves. After adding WEO, although some wavy grooves remain, the surface becomes more uniform, indicating improved homogeneity and consistency in the regenerated asphalt.
(4) The proportion of asphaltene and resin in aged asphalt increases, primarily due to the formation of carbonyl and sulfinyl groups. WEO replenishes the lost lightweight components of aged asphalt, especially increasing the alkanes and other hydrocarbons, which improves the asphalt’s overall performance.
(5) Molecular simulations indicated that aging reduced the diffusion abilities of Ar and S in the asphalt. However, upon the addition of WEO, the diffusion capacities of the lightweight components (Ar and S) were significantly enhanced, which is consistent with the viscosity index observed in the macroscopic tests.
(6) The use of WEO for regenerating waste asphalt aligns with the principles of a circular economy, effectively turning waste into a valuable resource. From an economic perspective, the regeneration process generates a profit of approximately 65,000 yuan per ton, demonstrating the feasibility of WEO as an asphalt regenerator both economically and environmentally.

The study provides a performance evaluation and mechanism analysis of the benefits of using WEO for restoring the performance of aged asphalt, and assesses the use of WEO from a socio-economic perspective. The results indicate that WEO has superior rejuvenating abilities for aged asphalt, significantly enhancing its performance. However, lighter oils like WEO may negatively impact the high-temperature performance of the asphalt. Subsequent research could focus on studying the changes in the high-temperature performance of aged asphalt.

## Supporting information

S1 Data Original data. (RAR)
